# Case report: Evaluation of myocardial microcirculation in patients with breast cancer after anthracycline chemotherapy by using intravoxel incoherent motion imaging

**DOI:** 10.3389/fcvm.2022.900309

**Published:** 2022-09-23

**Authors:** Shilan Li, Di Tian, Xin Li, Jia Li, Qingwei Song, Yunlong Xia, Zhiyong Li

**Affiliations:** ^1^Department of Radiology, The First Affiliated Hospital of Dalian Medical University, Dalian, China; ^2^Department of Oncology, The First Affiliated Hospital of Dalian Medical University, Dalian, China; ^3^Department of Cardiology, The First Affiliated Hospital of Dalian Medical University, Dalian, China

**Keywords:** breast cancer, anthracycline, cardiotoxicity, IVIM, microcirculation

## Abstract

**Introduction:**

Anthracycline chemotherapy drugs can produce cardiotoxicity in patients with breast cancer, leading to myocardial cell death and fibrosis, further developing into cardiac failure. However, the condition of myocardial microcirculation was unknown in breast cancer after anthracycline chemotherapy. As a result, intravoxel incoherent motion (IVIM) imaging was used to non-invasively observe the condition of myocardial microcirculation in a patient with breast cancer after anthracycline chemotherapy.

**Case report:**

A 43-year-old female patient with a right breast lump was reported. Preoperative ultrasound-guided needle biopsy showed invasive carcinoma of the right breast with fibroadenoma. Sentinel lymph node biopsy combined with simplified radical surgery for right breast cancer was performed. Postoperative pathological findings reported breast cancer (pT2N2M0 IIIA). The patient underwent eight sessions of the EC-TH chemotherapy scheme, and the EC and the TH schemes were adopted for the first four sessions and the last four sessions, respectively. During chemotherapy, during which there was the occurrence of Grade II myelosuppression, chest CT and abdomen CT showed no metastasis, and ECG and cardiac ultrasound reports returned to normal. Cardiac cine magnetic resonance and IVIM imaging were performed at the beginning of the first chemotherapy session (baseline) and after the third, fifth, and eighth chemotherapy sessions, respectively. We found that the fast apparent diffusion coefficient (ADC_fast_) and f parameters appeared to show a downward trend from the baseline to the fifth chemotherapy session, where the IVIM_fast_ values declined from 163 × 10^−3^ mm^2^/s to 148 × 10^−3^ mm^2^/s and finally to 134 × 10^−3^ mm^2^/s and f values declined from 45% to 36% and then to 30%, respectively. ADC_fast_ and f values showed an inclination from the fifth and eighth chemotherapy sessions.

**Conclusion:**

Our case report showed that IVIM technology can likely detect non-invasive myocardial microcirculation early and quantitatively after anthracycline chemotherapy in patients with breast cancer. That is, IVIM technology seems to be helpful for cardiovascular risk monitoring and prognosis assessment of myocardial microcirculation in patients with breast cancer after anthracycline chemotherapy.

## Introduction

Breast cancer is the most common malignant tumor in women, ranking as the highest incidence of cancer among women and the main cause of cancer-related deaths in women (1). Radical surgical treatment and postoperative adjuvant chemotherapy can greatly reduce breast cancer mortality. Anthracycline is a major component of first-line chemotherapy for breast cancer because it is a chemical substance with anti-tumor activity produced by microorganisms (2). However, anthracyclines induce cardiotoxicity, and early stages of cardiotoxicity are associated with inflammation, vacuolation, and edema of the myocardial cells, leading to myocardial cell fibrosis and cardiac failure (3–5). Early cardiotoxicity precedes cardiac function after chemotherapy in patients with breast cancer, which highlights the importance of early recognition of cardiac injury and the establishment of cardioprotective treatment mechanisms to prevent cardiac failure.

IVIM is a new non-invasive technology that can analyze the characteristics of the myocardial tissue from the perspective of microcirculation, and early cardiac toxicity after anthracycline chemotherapy in patients with breast cancer was observed by using the IVIM technique, providing a new idea for future research and hoping to be better applied in clinical practice in the future. Therefore, the IVIM technology was initially used to observe the laboratory parameters of patients with breast cancer after anthracycline chemotherapy, and a case regarding is reported below.

## Case report

**Figure 1:**
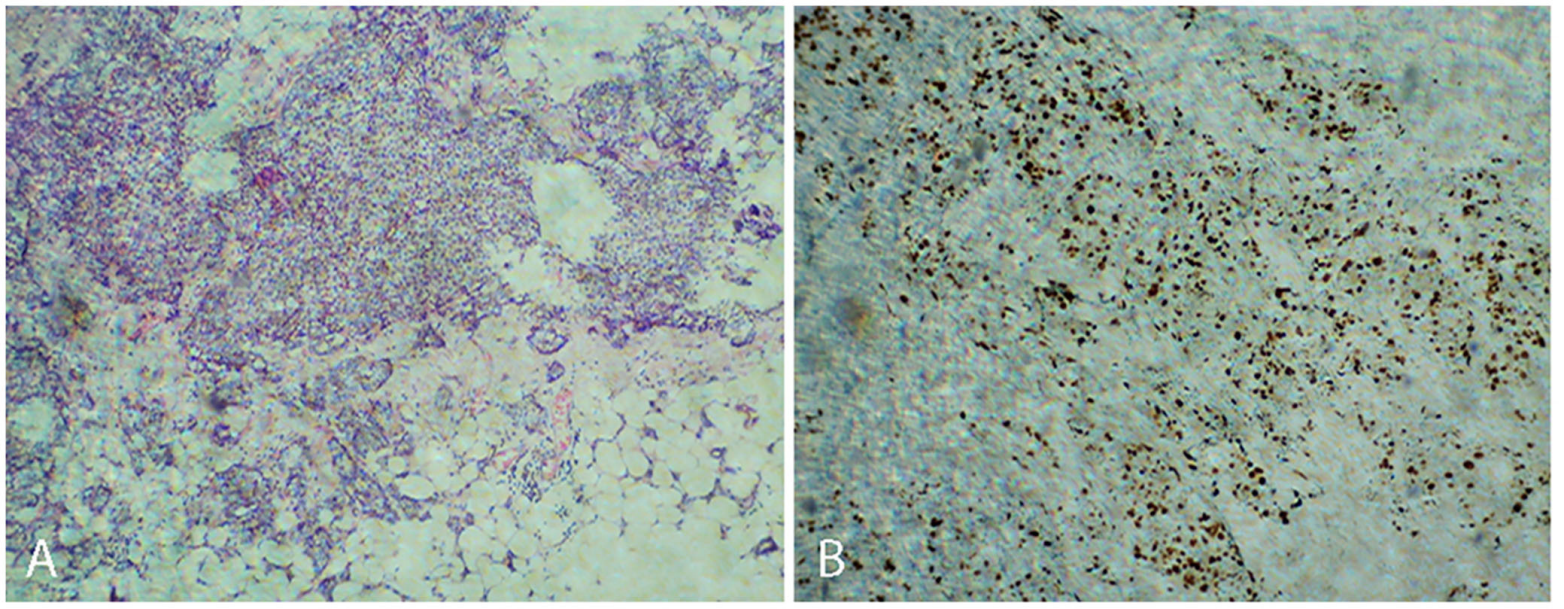
Non-specific invasive carcinoma of the right breast (invasive ductal carcinoma SBR II-III) **(A)**. Positive immunohistochemical staining for Ki 67 **(B)**.

A 43-year-old woman accidentally found a right breast lump on March 2014, with a diameter of 2 × 2 cm and stabbing pain. The mass was not related to the menstrual cycle. There was no redness, swelling, or rupture of the skin near the lump. No erosion, stabbing pain, pruritus, or discharge of the nipple was observed. In 2016, the tumor became progressively enlarged, and a mass of 3 × 2 cm was found under the right axilla. In March 2017, there was pain in the right axilla with obvious tenderness. Physical examination determined with touch indicated a tough mass of 5 × 3 cm in the right breast (between 7 and 9 o'clock), and the lump was characterized by unpolished surface, obscure boundary, and poor activity. A soft mass of 4 × 2 cm was touched in the right axilla, and no obvious abnormality was found during the rest of the physical examination. Ultrasound examination suggested multiple solid masses in the right breast. The dimensions of the tumor determined between 6 and 11 o'clock were 5.3 × 3.4 cm, which was classified as BI-RADS 4C-5; the dimensions of the mass identified at 10 o'clock were 1.2 × 0.5 cm, which was classified as BI-RADS 4a. The dimensions of enlarged lymph nodes in the right axilla were 1.2 × 0.6 cm. Ultrasound-guided needle biopsy showed an invasive carcinoma of the right breast with fibroadenoma. Surgical treatment was performed on 9 March 2017. Intraoperative sentinel lymph node biopsy found metastatic cancer, and simplified radical mastectomy was performed for right breast cancer. Postoperative pathology showed non-specific invasive carcinoma of the right breast (invasive ductal carcinoma SBR II-III) and mucinous carcinoma of high to medium grade (intraductal carcinoma) of the dimension 3.5 × 1.5 × 3.0 cm, as seen in Figure 1A. The other three lesions were non-special invasive carcinoma (invasive ductal carcinoma SBR II), with the dimensions of 0.7 × 0.7 × 0.5 cm, 1.0 × 0.6 × 0.5 cm, and 1.0 × 0.8 × 0.5 cm. No metastasis was found in the right axillary lymph node (0/15). Positive immunohistochemical staining for ER, PR, HER-2, AR, P53, and Ki 67 (Figure 1B) was performed. The postoperative stage was pT2N2M0 IIIA, Lumina I B. The chemotherapy regimen was EC-TH chemotherapy, with 8 sessions of chemotherapy completed from April 7, 2017 to September 25, 2017.

During the follow-up, corresponding examinations were made according to the patient's condition. Between the baseline and eighth chemotherapy sessions, ECG, cardiac ultrasonography and breast ultrasound, chest CT and upper abdomen CT, and ECT were performed in the following order: Chest CT, upper abdomen CT, and cardiac ultrasound were performed at baseline; ECG examination was performed after the first chemotherapy and the third chemotherapy. On the fifth chemotherapy session, none of the above examinations were performed. Chest CT, upper abdomen CT, cardiac ultrasonography, and breast ultrasound were performed during the eight chemotherapy session. Breast ultrasound results showed (1) a right breast surgery, (2) multiple cystic nodules in the left breast, and (3) no enlarged lymph nodes under both axilla and supraclavicular, and the rest of the examination results were normal. Degree II myelosuppression occurred during chemotherapy, and hematology returned to normal after treatment with granulocyte colony-stimulating factor (G-CSF).

**Figure 2:**
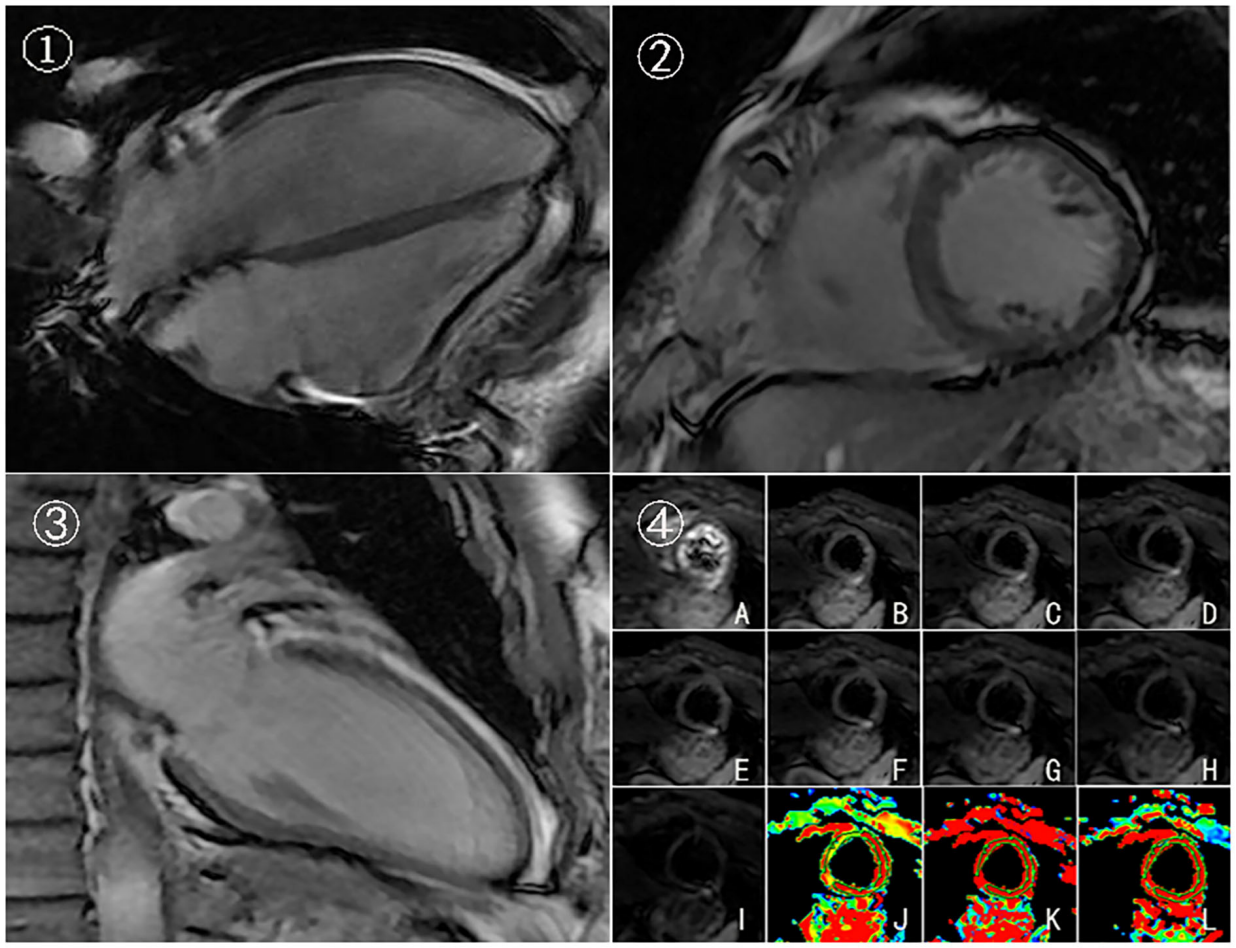
Cine images and IVIM images of baseline. 4-chamber(➀), left ventricle (LV) short-axis ➁, 2-chamber➂, and IVIM images ➃ of baseline. (A–I) Original images of IVIM at the left ventricular short-axis view (corresponding to b = 0, 20, 50, 80,100, 120, 200, 300, and 500 s/mm^2^, respectively). (J–L) Pseudocolor images were produced using the bi-exponential mode of the IVIM imaging system (corresponding to ADC_slow_, ADC_fast_, and f values, respectively).

**Figure 3:**
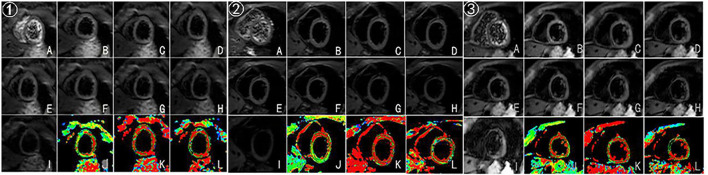
(A–I) Original images of IVIM at the left ventricular short-axis view (corresponding to b = 0, 20, 50, 80,100, 120, 200, 300, and 500 s/mm^2^, respectively). (J–L) Pseudocolor images were produced using the bi-exponential mode of the IVIM imaging system (corresponding to ADC_slow_, ADC_fast_, and f values, respectively). ➀, ➁, ➂ represent IVIM images after the third, fifth, and eighth chemotherapy sessions.

In this case, CMR examinations were performed at the beginning of the first chemotherapy (baseline) and after the third, fifth, and eighth chemotherapy sessions, using a 3.0 T magnetic resonance imager (platform HDxt; General Electric Medical Systems, Waukesha, WI) equipped with an 8-channel phased-array cardiac coil. Standard 2-, 3-, and 4-chamber and left ventricle (LV) short-axis cine images from apical to basal were acquired with fast imaging employing a steady-state acquisition sequence. IVIM imaging was performed with the echo planar imaging (EPI) sequence. LV structural and functional parameters were measured by the Qmass package (Medis^®^ Suite MR), as seen in Table 1. IVIM parameters were obtained by using GE Functool 9.4.05a software, as seen in Table 2. Cine images of 4-chamber, 2-chamber, left ventricle (LV) short-axis and IVIM images of baseline are shown in Figure 2. IVIM images of the third, fifth, and eighth chemotherapy sessions are shown in Figure 3.

**Table 1:** The situation at the baseline and after the third, fifth, and eighth chemotherapy sessions.

	**Baseline**	**Third**	**Fifth**	**Eighth**
Left atrium (anterior and posterior diameter, cm)	6.21	6.73	6.45	6.54
Right atrium (vertical atrial septum, cm)	6.12	6.67	6.47	6.58
Left ventricular transverse diameter (cm)	4.73	6.81	7.12	6.71
Right ventricular transverse diameter (cm)	4.93	5.12	5.20	5.11
LVEF (%)	56.91	62.48	63.16	71.70
LVEDV (ml)	128.31	151.82	136.88	160.94
LVESV (ml)	55.30	56.97	50.42	61.64
CO (L)	4.53	6.07	4.76	6.16
LV ED mass (g)	65.49	78.4	73.17	84.91
BMI (kg/m^2^)	24.8	26.98	26.98	26.98

**Table 2:** IVIM parameters at the baseline and after the third, fifth, and eighth chemotherapy sessions.

	**Baseline**	**Third**	**Fifth**	**Eighth**
ADC_slow_ (× 10^−3^ mm^2^/s)	2.88	2.25	2.55	3.5
ADC_fast_ (× 10^−3^ mm^2^/s)	163	148	134	171
f (%)	45%	36%	30%	31%

## Discussion

Water molecules include water molecules both at the intracellular and intercellular levels and at the intravascular level in the human body. However, diffusion-weighted imaging (DWI) cannot distinguish microcirculation perfusion and extravascular dispersion by using a single b-value model. Le Bihan first proposed the intravoxel incoherent motion technique for reflecting the movement of water molecules in tissues and microcirculation (6). With the progress of IVIM technology in sequence design and data processing (7–9), Callot et al. (10) attempted to use IVIM imaging on living animals and found it to be feasible. The technology observes the movement of water molecules in tissue cells and microvessels without any contrast agent, and ADC_fast_ and f parameters reflect the condition of microcirculation perfusion. Further, Delattre et al. (11) and Mou et al. (12) applied it to the human body and proved that it was feasible.

Using the ICC test, Mou et al. (12) found that the IVIM parameters were consistent in intra-observer and inter-observer values in 30 healthy volunteers (at least one slice successful acquisition). This study found that the intra- and inter-observer consistencies of ADC_slow_, ADC_fast_, and f values were 0.87, 0.89, 0.93, 0.97, 0.93, and 0.96, respectively. In addition, Moulin et al. (13) evaluated the inter-measurement reproducibility of IVIM parameters by Lin's concordance coefficient and the Bland-Altman analysis on 10 healthy volunteers. In this study, f values and ADC_slow_ showed high repeatability (Pc = 0.963; Pc = 0.762) and ADC_fast_ showed low repeatability (Pc = 0.652). Li found that intra-observer and inter-observer consistencies were good in 80 healthy volunteers (14).

Cardiotoxicity induced by anthracyclines was first proposed by Lefrak et al. (15). Cardiotoxicity includes arrhythmias, cardiac failure, and myocardial injury. It may occur months to years after the completion of primary treatment and can severely impair life quality and overall survival of the patient. Besides, it can be classified as acute, chronic, and delayed (16). Cardiotoxicity from anthracyclines is a dose-dependent toxicity, the risk of which increases exponentially with cumulative doses, which irreversibly results in myocardial cell structural changes and cell death in the chronic and delayed stages (17). Therefore, early and effective detection of cardiotoxicity of anthracyclines is an effective means to guide clinical medication and minimize cardiotoxicity. CMR is the gold standard for evaluating cardiac function due to its multi-mode imaging and high repeatability. CMR has high sensitivity and accuracy for cardiac injury caused for various reasons, especially in the early stage. In the early stages of cardiac injury, CMR can detect edema, inflammation, and fibrosis of cardiomyocytes, playing an important role in the diagnosis of early cardiotoxicity in patients with cancer (18). However, so far, no studies were reported on myocardial microcirculation after chemotherapy for patients with cancer using IVIM imaging.

In this case, the EC-TH (Epirubicin, Cyclophosphamide, Docetaxel, Trastuzumab) scheme was used. The selection of EC-TH, in this case, was related to the patient's postoperative pathology. Since lymph node metastasis occurred in the pathology of this case, postoperative adjuvant chemotherapy was required, so EC-TH was selected according to the recommendation of international guidelines (19). The details of medication are as follows: EPI(Epirubicin) 70 mg d1-2 ivgtt and CTX(Cyclophosphamide) 900 mg d1 ivgtt Q21d before the fifth chemotherapy. The TH regimen was used from the fifth to the eighth chemotherapy sessions, and the details of the medication are as follows: TXT, 160 mg and Herceptin, 500 mg ivgtt.

In this case, baseline and the third, fifth, and eighth sessions of chemotherapy were selected because, after the first and second chemotherapy sessions, the time of drug action on the myocardium was shorter, so IVIM was chosen after the third chemotherapy to observe the myocardial microcirculation. Besides, the choice of the fifth chemotherapy is related to the chemotherapy regimen of this case, the EC-TH was used in this case, anthracycline was used for the first four sessions, and docetaxel and trastuzumab were used for the last four sessions in order to observe the effect of drugs on cardiac microcirculation. The eighth session was observed for the overall situation after chemotherapy. The early stages of cardiotoxicity include cellular vacuolation and edema, which could lead to microcirculation dysfunction; however, the IVIM technology can be used to observe the early stage of cardiotoxicity from the perspective of microcirculation. ADC_fast_ and f values showed a downward trend from the baseline to the fifth chemotherapy, and it may be because anthracycline was used before the fifth chemotherapy, so the case showed that anthracycline causes damage to myocardial microcirculation. In terms of histological features, early stages of cardiotoxicity include cellular vacuolation and edema, which could lead to microcirculation dysfunction. In addition, ADC_fast_ and f values showed an upward trend from the fifth chemotherapy to the eighth chemotherapy session in the case. Possible reasons for this could be the following: early myocardial damage may be recovered after the withdrawal of anthracyclines, because cardiotoxicity of anthracyclines can be classified into acute, chronic, and delayed according to time. Among these classifications of cardiac damage, the acute is reversible, and clinical manifestations of chronic and delayed cardiotoxicity will manifest as cardiac failure and cardiac dysfunction (20); however, the cardiac function of the case remained normal from baseline to the eighth chemotherapy, suggesting that the patient may be in early myocardial toxicity rather than a phase of chronic and delayed cardiotoxicity. In addition, due to individual differences and because the case was of a middle-aged woman without cardiovascular disease, good physical and mental conditions were observed. The specific reasons remain to be studied.

## Conclusion

Anthracyclines can induce cardiotoxicity. Early stages of cardiotoxicity include cellular vacuolation and edema, which could lead to microcirculation dysfunction. However, CMR is the gold standard for evaluating cardiac structure and function, and the IVIM technology is likely to non-invasively observe early myocardial microcirculation, that is, early and effective detection of anthracycline cardiotoxicity can help to guide clinical medication to minimize cardiotoxicity and improve overall survival.

## Data availability statement

The original contributions presented in the study are included in the article/supplementary material, further inquiries can be directed to the corresponding author.

## Ethics statement

The studies involving human participants were reviewed and approved by Ethics Committee of the First Affiliated Hospital of Dalian Medical University(PJ-KS-KY-2022-69). Written informed consent to participate in this study was provided by the participants' legal guardian/next of kin. Written informed consent was obtained from the individual(s) for the publication of any potentially identifiable images or data included in this article.

## Author contributions

All authors listed have made a substantial, direct, and intellectual contribution to the work and approved it for publication.

## Conflict of interest

The authors declare that the research was conducted in the absence of any commercial or financial relationships that could be construed as a potential conflict of interest.

## Publisher's note

All claims expressed in this article are solely those of the authors and do not necessarily represent those of their affiliated organizations, or those of the publisher, the editors and the reviewers. Any product that may be evaluated in this article, or claim that may be made by its manufacturer, is not guaranteed or endorsed by the publisher.
